# Real-World Prescribing Patterns of SGLT2 Inhibitors and GLP-1 Receptor Agonists in Older Adults with Type 2 Diabetes and Cardiometabolic Disease

**DOI:** 10.3390/ph19010009

**Published:** 2025-12-20

**Authors:** Ibrahim S. Alhomoud, Khalid A. Alamer

**Affiliations:** 1 Department of Pharmacy Practice, College of Pharmacy, Qassim University, Qassim 51452, Saudi Arabia; 2 Department of Pharmacy Practice, College of Pharmacy, Imam Abdulrahman Bin Faisal University, Dammam 31441, Saudi Arabia; kaalamer@iau.edu.sa

**Keywords:** diabetes mellitus, SGLT2 inhibitors, GLP-1 receptor agonists, older adults, cardiovascular risk, underutilization

## Abstract

**Background/Objectives:** Older adults with type 2 diabetes frequently have cardiovascular or kidney disease, and current guidelines strongly recommend the use of SGLT2 inhibitors or GLP-1 receptor agonists in these high-risk populations. This study aimed to critically evaluate their real-world utilization in a tertiary care setting to identify gaps in prescribing and opportunities for improvement. **Methods:** A retrospective cross-sectional analysis was conducted using electronic medical records from a tertiary academic hospital in Saudi Arabia (June 2019–May 2023). Patients aged ≥65 years with type 2 diabetes and documented ASCVD, heart failure, or CKD were classified as guideline-eligible. Prescribing rates, trends, specialty variation, and associated factors were assessed. **Results:** Among 223 older high-risk patients with type 2 diabetes, 83.4% received an SGLT2 inhibitor or GLP-1 receptor agonist. However, only 1.6% (223 out of 14,146 older adults) were identified in the electronic medical record as having ASCVD, heart failure, or CKD, suggesting potential underdiagnosis or incomplete recognition of guideline-eligible comorbidities. Overall, just 7.4% of older adults with type 2 diabetes were prescribed either therapy. Notably, approximately one-quarter of prescriptions originated from specialties not routinely involved in cardiometabolic care, indicating variability in prescribing patterns. Multivariable analysis showed that older age, female sex, and unmarried status were associated with lower odds of receiving therapy, while patients seen in cardiology or internal medicine had higher odds than those seen in primary care. **Conclusions:** Prescribing of GLP-1 receptor agonists and SGLT2 inhibitors was aligned with guideline recommendations in documented high-risk patients. Nevertheless, overall utilization remained low, indicating gaps in the recognition of cardiometabolic comorbidities. Enhancing routine cardiovascular and kidney risk assessment may improve therapy optimization.

## 1. Introduction

The high global prevalence of diabetes mellitus increases the medical and economic burden on healthcare systems. More than 500 million people of all ages currently live with diabetes mellitus [1]. Global projections estimate that this number could reach 1.3 billion by 2050 [1]. The burden is particularly pronounced in the older adult population, with prevalence increasing with age and reaching approximately 30% among those 65 years or older in recent United States surveillance data [2]. In Saudi Arabia, a recent nationwide survey of individuals aged 60 years and older found that 44.7% were diagnosed with type 2 diabetes, indicating that nearly one in two older adults live with the disease [3].

Diabetes mellitus contributes to atherosclerosis and atherothrombosis through multiple mechanisms, including chronic inflammation, endothelial dysfunction, and oxidative stress [4]. Large registry studies from the United States have shown that approximately 50% of individuals with type 2 diabetes have established atherosclerotic cardiovascular disease (ASCVD) [5,6]. This burden of cardiovascular morbidity and mortality is particularly pronounced among older adults. The American Diabetes Association (ADA) recommends using sodium–glucose cotransporter-2 (SGLT2) inhibitors and glucagon-like peptide-1 (GLP-1) receptor agonists with proven cardiovascular benefits in individuals with type 2 diabetes and established ASCVD. As additional evidence has demonstrated kidney and heart failure benefits of these therapies, guideline recommendations have subsequently expanded to include these high-risk populations [7,8,9]. Other major clinical guidelines have also similarly endorsed the use of these agents irrespective of glycated hemoglobin (A1C) levels [10,11,12,13,14]. Despite increased utilization of SGLT2 inhibitors and GLP-1 receptor agonists among individuals with type 2 diabetes in recent years, many eligible individuals continue to go untreated with these therapies in routine clinical practice [15,16,17,18,19,20,21,22,23].

Robust evidence from cardiovascular outcome trials supports the use of cardioprotective antihyperglycemic therapies in this population. A pooled analysis of 93,502 participants from 11 cardiovascular outcome trials demonstrated that among patients aged ≥65 years with type 2 diabetes, GLP-1 receptor agonists reduced major adverse cardiovascular events (MACE) by 14% (HR 0.86; 95% CI, 0.80 to 0.92) [24]. Similarly, SGLT2 inhibitors reduced MACE by 10% (HR 0.90; 95% CI, 0.83 to 0.98), reduced hospitalizations for heart failure by 38% (HR 0.62; 95% CI, 0.51 to 0.76), and slowed kidney disease progression by 43% (HR 0.57; 95% CI, 0.43 to 0.77) [24]. Subgroup analyses further demonstrated that patients aged ≥75 years experienced comparable benefits, demonstrating that the efficacy of these agents is maintained even at advanced age [24]. Real-world data increasingly demonstrate that SGLT2 inhibitors remain effective and well-tolerated even among very old adults. In the AGING-HF observational study of 496 patients hospitalized with acute heart failure, with a mean age of 90 years and a high comorbidity burden (mean Charlson score 8.2), SGLT2 inhibitor use was associated with lower all-cause mortality (HR 0.67; 95% CI, 0.46 to 0.98) and fewer heart failure rehospitalizations (HR 0.64; 95% CI, 0.42 to 0.97) over one year [25]. Despite advanced age and multimorbidity, treatment discontinuation remained low (2.7%) [25]. Additional emerging evidence suggests that these therapies may provide benefits in older adults with type 2 diabetes beyond cardiometabolic effects [9,26]. In a Medicare-based cohort of more than 52,000 older adults, initiation of GLP-1 receptor agonists or SGLT2 inhibitors was associated with significantly slower frailty progression compared with DPP-4 inhibitors, with mean 1-year Frailty Index improvements of −0.0063 and −0.0057, respectively (both *p* < 0.01) [26].

Real-world evidence on the use of GLP-1 receptor agonists and SGLT2 inhibitors in older individuals (≥65 years) remains limited, despite this population having a higher burden of cardiovascular and kidney disease. Additionally, older individuals are often underrepresented in clinical research, which has led to gaps in understanding real-world treatment patterns in this high-risk population. Therefore, this study focuses on older individuals to examine the real-world utilization of GLP-1 receptor agonists and SGLT2 inhibitors.

## 2. Results

### 2.1. Demographic and Clinical Characteristics

**Figure 1:**
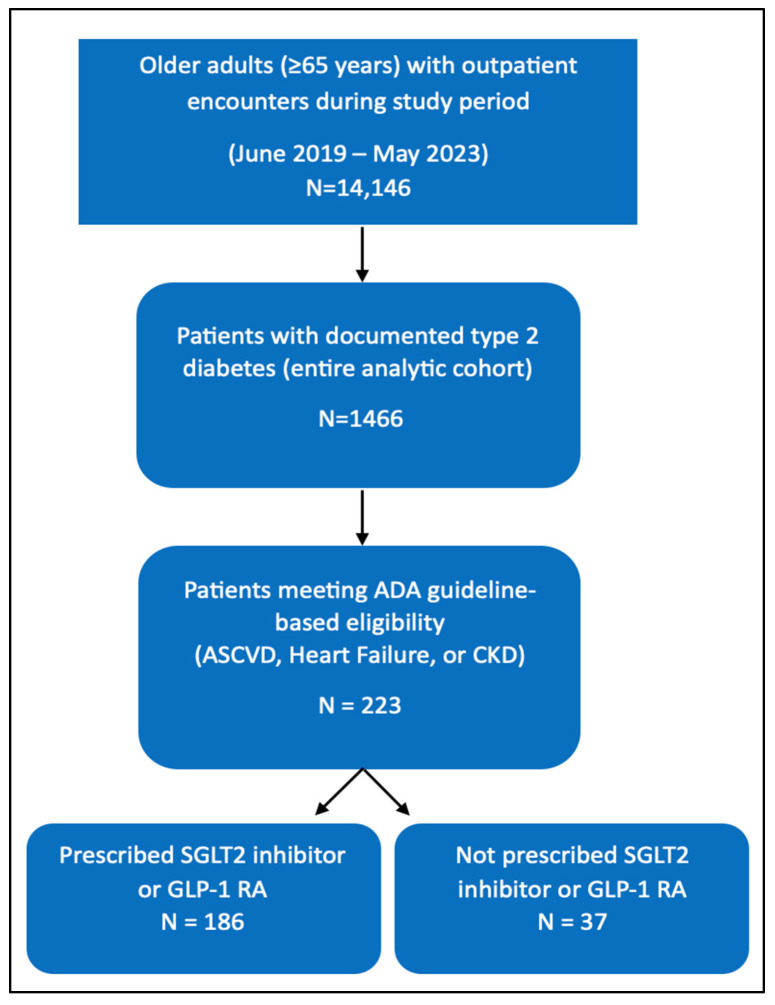
Study population flow diagram.

A total of 223 patients aged ≥65 years with type 2 diabetes and either ASCVD, heart failure, or CKD were included in the analysis. The selection of the study population is summarized in Figure 1. The mean age of the cohort was 74.92 years (SD, 6.23), and 42.15% were female. Established ASCVD was the most prevalent comorbidity, observed in 93.72% of patients, while heart failure and CKD were less common, present in 4.04% and 3.59%, respectively.

Throughout the Results section, two analytic populations are described. The “full outpatient cohort” includes all older adults aged ≥65 years who attended outpatient care during the study period (N = 14,146). The “guideline-eligible subgroup” consists of patients who met ADA-recommended criteria for SGLT2 inhibitor or GLP-1 receptor agonist therapy because of documented ASCVD, heart failure, or CKD (N = 223).

Among those prescribed a GLP-1 receptor agonist or SGLT2 inhibitor, the mean age was 73.55 years (SD, 6.04), and females represented 39.40% of patients, compared with 75.38 years (SD, 7.74) and 49.28% in the non-prescribed group. In the prescribed group, the prevalence of ASCVD, heart failure, and CKD was 1.22%, 0.05%, and 0.05%, respectively, compared with 4.99%, 0.18%, and 0.28% in those who were not prescribed either therapy. A summary of patient demographics, sex distribution, and comorbidity prevalence by treatment group is presented in Table 1. Additional details on the distribution of individual SGLT2 inhibitors and GLP-1 receptor agonists stratified by ASCVD, heart failure, and CKD are provided in the Supplementary Material (Supplementary Table S1).

Table 1 displays characteristics from two distinct analytic populations. The “Prescribed” (N = 1052) and “Not Prescribed” (N = 13,094) columns reflect the full outpatient cohort of older adults aged ≥65 years (total N = 14,146). In contrast, the “Total Eligible” column (N = 223) represents only the subset of older adults with type 2 diabetes who had documented ASCVD, heart failure, or CKD and therefore met guideline-based eligibility criteria.

### 2.2. Primary Outcome: Overall Prescribing Rate

Among all older adult outpatient cohorts (N = 14,146 encounters/unique patients included in the analytic file), a total of 1052 individuals received at least one dispensing of a GLP-1 receptor agonist or an SGLT2 inhibitor during the study window, corresponding to a population-level dispensing proportion of 7.4% (Table 2A).

Within the guideline-eligible subgroup, we identified 223 eligible patients, of whom 186 received either a GLP-1 receptor agonist or an SGLT2 inhibitor. This corresponds to a prescribing rate of 83.4% among eligible patients (Table 2B).

Table 2A presents prescribing patterns in the whole outpatient cohort (N = 14,146), whereas Table 2B presents prescribing patterns in the guideline-eligible subgroup (N = 223). These denominators represent different populations and are not expected to match.

### 2.3. Prescribing Patterns Among Guideline-Eligible Older Adults Stratified by Comorbidity

Prescribing patterns were further examined according to comorbidity subgroup (Table 3). Among the 209 patients with ASCVD, 172 received a GLP-1 receptor agonist or an SGLT2 inhibitor, which represented a prescribing rate of 85.3%. In the heart failure and CKD subgroups, 7 of 9 patients (77.8%) and 7 of 8 patients (87.5%), respectively, were prescribed therapy.

Table 3 exclusively reports prescribing patterns within the guideline-eligible subgroup (N = 223), stratified by comorbidity.

### 2.4. Patterns of Prescribing GLP-1 Receptor Agonists and SGLT2 Inhibitors over Time Among Older Adults (2019–2023)

**Figure 2:**
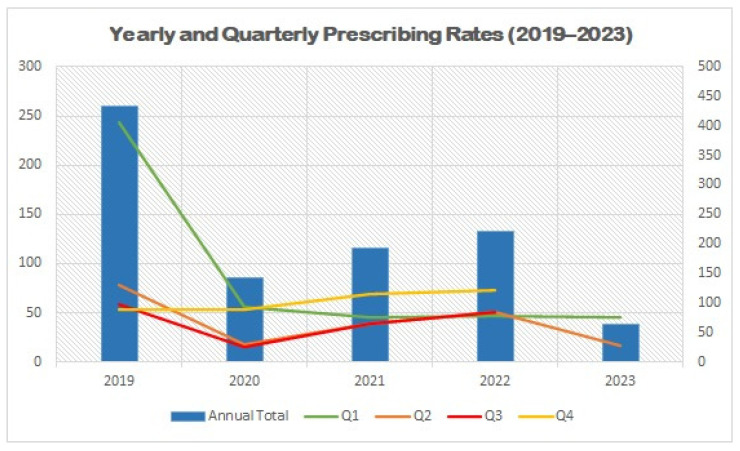
Yearly and Quarterly Prescribing Rates of GLP-1 Receptor Agonists and SGLT2 Inhibitors Among Older Adults (2019–2023). Abbreviations: Q1–Q4 = calendar year quarters.

Analysis of yearly and quarterly prescribing trends between 2019 and 2023 demonstrated marked variability in the uptake of GLP-1 receptor agonists and SGLT2 inhibitors among older adults with type 2 diabetes (Table 4, Figure 2). Prescribing was highest in 2019, when 432 patients (3.05% of the cohort) were initiated on therapy. However, a substantial decline was observed in 2020, with the annual prescribing rate dropping to 1.0% (142 patients). Prescribing remained relatively low during 2021 (1.36%) and showed modest recovery in 2022 (1.57%), followed by a further decline in 2023 (0.45%).

Quarterly trends were consistent with the annual fluctuations, with the highest quarterly prescribing rates observed in Q1 of 2019, followed by sharp reductions across subsequent quarters in 2020 and 2021. Although a modest rebound occurred in 2022, prescribing rates never returned to the levels observed before 2020.

### 2.5. Prescribing Patterns by Specialty

Prescribing patterns varied substantially across medical specialties (Table 5). The highest prescribing rate was observed among patients followed in internal medicine, where 11 of 16 eligible patients (68.8%) received a GLP-1 receptor agonist or an SGLT2 inhibitor. Cardiology and primary care showed comparable but lower prescribing rates, with 44.3% (31 of 70) and 42.6% (52 of 122) of eligible patients prescribed therapy, respectively. In contrast, prescribing in other departments was less frequent, with only 26.7% (4 of 15) of patients receiving treatment.

### 2.6. Factors Associated with Prescribing SGLT2 Inhibitors or GLP-1 Receptor Agonists Among Guideline-Eligible Older Adults

Logistic regression analyses further explored factors associated with prescribing (Table 6). In univariate analyses, age was inversely associated with prescribing (OR 0.964; 95% CI 0.955–0.974; *p* < 0.001), indicating that older age was associated with a reduced likelihood of receiving therapy. Specialty also played a significant role: compared with primary care (reference group), patients followed in cardiology (OR 1.723; 95% CI 1.195–2.484; *p* = 0.004) and medicine (OR 1.419; 95% CI 1.112–1.811; *p* = 0.005) had higher odds of being prescribed therapy, whereas those in other specialties had markedly lower odds (OR 0.399; 95% CI 0.314–0.507; *p* < 0.001).

In the multivariate model adjusting for covariates, these associations remained significant: cardiology (OR 1.572; 95% CI 1.059–2.331; *p* = 0.025) and medicine (OR 1.448; 95% CI 1.116–1.878; *p* < 0.005) were independently associated with higher prescribing rates, while other specialties continued to show lower odds (OR 0.399; 95% CI 0.309–0.516; *p* < 0.001). Patient sex and marital status also emerged as significant predictors in the overall cohort. Women (OR 0.715; 95% CI 0.622–0.820; *p* < 0.001) and unmarried individuals (OR 0.663; 95% CI 0.551–0.798; *p* < 0.001) were less likely to be prescribed therapy compared with men and married patients, respectively.

### 2.7. Prescribing Patterns Stratified by Specialty Across Comorbidity Subgroups

Given the high burden of comorbidities among older adults with type 2 diabetes, prescribing rates were further evaluated within subgroups of patients with ASCVD, heart failure, and CKD, stratified by specialty (Table 7, Table 8 and Table 9). For patients with ASCVD, specialty was a significant factor in prescribing likelihood. In the univariate analysis, cardiology and medicine specialties with primary care. However, these associations were attenuated in the multivariate analysis. Within the ASCVD subgroup, patient sex remained an independent predictor. Women were significantly less likely to be prescribed therapy compared with men (OR 0.555; 95% CI 0.371–0.972; *p* = 0.040).

Among patients with heart failure (Table 8), neither age, specialty, marital status, nor sex demonstrated a statistically significant association with prescribing after adjustment, likely reflecting the small subgroup size and limited prescribing events. Similarly, in patients with CKD (Table 9), no independent predictors of prescribing were identified in multivariable analysis, although cardiology demonstrated a numerically higher odds ratio (OR 5.273; 95% CI, 0.161–8.649; *p* = 1.000).

## 3. Discussion

This study provides novel real-world insights into the prescribing patterns of SGLT2 inhibitors and GLP-1 receptor agonists among older individuals (≥65 years) with type 2 diabetes and established cardiovascular or kidney disease. At a population level, uptake of these therapies was modest, with 7.4% of all older adults in the outpatient cohort receiving either agent. However, when examining individuals who met guideline-based eligibility criteria (i.e., those with established ASCVD, heart failure, or CKD), prescribing rates were substantially higher. Among the 223 eligible older individuals identified, 83.4% received either an SGLT2 inhibitor or a GLP-1 receptor agonist. This high adoption rate indicates that, once patients are correctly identified as high risk, clinicians in this setting largely adhere to contemporary guideline recommendations. While awareness of SGLT2 inhibitors and GLP-1 receptor agonists is generally high among physicians in Saudi Arabia, gaps in training and consistent application of clinical guidelines persist, particularly in primary care settings [27]. Endocrinologists tend to demonstrate greater confidence and consistency in prescribing these therapies, likely due to their specialized experience and familiarity [28]. Moreover, access and formulary availability have improved, especially following the introduction of generic SGLT2 inhibitors, which are expected to enhance affordability and facilitate broader use among guideline-eligible patients.

The higher prescribing rate observed in our study compared with prior real-world reports is likely attributable to differences in population characteristics and clinical care pathways. Our analysis focused specifically on older adults (≥65 years) with documented ASCVD, heart failure, or CKD receiving care in a tertiary academic center, where cardiometabolic risk management is likely more emphasized. However, earlier studies evaluated broad adult cohorts across diverse healthcare settings [15,28,29,30,31]. For example, Dixon et al. reported that among adults with type 2 diabetes and cardiometabolic comorbidities receiving outpatient care, only 17.4% of eligible patients were prescribed either an SGLT2 inhibitor or a GLP-1 receptor agonist [15]. Additionally, data from a national U.S. cohort of over 759,000 patients with heart failure showed that only 10.1% overall received an SGLT2 inhibitor, with rates increasing from 4.6% in 2019 to 16.2% by 2023, yet still far below guideline expectations [29]. A study from Saudi Arabia reported that only 19% of adults with type 2 diabetes and cardiometabolic conditions meeting eligibility criteria received an SGLT2 inhibitor or a GLP-1 receptor agonist [28].

Our data further suggest that the relatively small proportion of patients identified as guideline-eligible may itself reflect a broader gap in disease recognition. Among the 14,146 older adults included in the cohort, only 223 (1.6%) had a documented diagnosis of ASCVD, heart failure, or CKD that would qualify them for therapy under contemporary guidelines. This prevalence is substantially lower than what would be expected based on the known epidemiology of these conditions among older individuals with type 2 diabetes, in whom rates of ASCVD and CKD are considerably higher. Although national epidemiological data on the prevalence of chronic diseases among older adults in Saudi Arabia remain limited, recent nationwide survey findings indicate that approximately 45% of individuals aged 60 years and older are living with type 2 diabetes [3]. In the CAPTURE study, documented ASCVD was reported in 15.1% of adults with type 2 diabetes in Saudi Arabia, which is higher than the prevalence in our cohort [32]. This discrepancy indicates that the main barrier observed in this cohort was the under-recognition or incomplete documentation of cardiovascular and kidney comorbidities that determine eligibility for guideline-directed therapy [33,34]. Despite national health initiatives to promote uptake of preventive care services, the actual use of routine screening remains limited. Only 23% of individuals aged 15 years or older in Saudi Arabia reported receiving a periodic health examination in the previous two years, while the majority of healthcare visits were prompted by acute illness or injury [35]. Addressing these barriers through proactive and standardized annual risk assessment procedures may therefore be essential to expanding appropriate use of GLP-1 receptor agonists and SGLT2 inhibitors among eligible older adults.

Approximately one-quarter of prescriptions for GLP-1 receptor agonists and SGLT2 inhibitors among guideline-eligible older adults originated from specialties outside internal medicine, cardiology, and primary care. Prescribing rates varied considerably across specialties, ranging from 26.7% in other fields to 68.8% in internal medicine (Table 5). This variation likely reflects differences in clinical scope, confidence in initiating and managing these therapies, and the extent of involvement in the care of patients with cardiovascular or kidney comorbidities. For older adults, this is particularly relevant, as they often require closer follow-up due to their increased susceptibility to adverse effects associated with these therapies, including volume depletion, kidney function fluctuations, and gastrointestinal intolerance [36]. When prescribing occurs across multiple specialties, opportunities for systematic follow-up, appropriate monitoring, dose titration, and patient counseling may be limited. Strengthening interdisciplinary coordination through shared care protocols, structured referral pathways, or pharmacist-supported follow-up may ensure that the use of these therapies is guideline-concordant and clinically safe for this high-risk population [37,38,39,40].

This study offers novel, real-world insights into the prescribing patterns of SGLT2 inhibitors and GLP-1 receptor agonists among older adults (≥65 years) with type 2 diabetes in Saudi Arabia. It specifically focuses on a high-risk population that is often underrepresented in clinical research yet more susceptible to cardiometabolic adverse outcomes. The application of current clinical guidelines to determine eligibility of participants enhanced the accuracy of identifying treatment gaps in high-risk individuals. The study also identifies specialty-level variation that may inform targeted interventions to improve uptake where needed. However, several considerations should be noted when interpreting these findings. As the study was conducted at a single tertiary care center, the results may not fully capture prescribing patterns in other healthcare settings. Although based on routinely documented diagnoses, eligibility estimates may be conservative due to potential underdocumentation of cardiovascular or kidney comorbidities. This limitation may have been further influenced by the COVID-19 pandemic, which may have affected healthcare access, documentation practices, and clinical decision-making related to prescribing patterns in this high-risk population. Another consideration is that while SGLT2 inhibitors and GLP-1 receptor agonists were analyzed collectively to reflect shared guideline indications, this approach may have masked class-specific trends. Finally, while the study does not evaluate prescribing rationale or clinical outcomes, it offers an important foundation for understanding the real-world adoption of these agents in a high-risk population. Future research should integrate pre–post or cohort-based designs to assess prescribing behaviors and associated cardiometabolic outcomes over time.

## 4. Materials and Methods

### 4.1. Study Design and Reporting

We conducted a retrospective cross-sectional study using routinely collected electronic medical record (EMR) data from a single academic medical center in Saudi Arabia. Reporting adheres to the STROBE guideline. This study was approved by the Institutional Review Board of Imam Abdulrahman Bin Faisal University, Dammam, Saudi Arabia (IRB-2025-05-0619).

### 4.2. Setting and Data Source

Data were extracted from the electronic medical record (EMR) of King Fahad University Hospital in Khobar, Saudi Arabia, a tertiary academic medical center affiliated with Imam Abdulrahman Bin Faisal University. The dataset included outpatient encounters from 1 June 2019 to 31 May 2023. Available fields included patient demographics (medical record number [MRN], date of birth, gender, nationality, marital status), visit/department details (e.g., internal medicine), diagnoses (ICD-10 code, diagnosis description, and onset date), and medication information from the pharmacy dispensing file (product identifier and description, dispense date, quantity dispensed, and prescriber identifier). Data from these modules were linked via direct linkage using each patient’s unique MRN. Before analysis, all dates were standardized, duplicate entries were removed, and a random subset of merged records was manually cross-checked against the source EMR to ensure data integrity and linkage accuracy.

### 4.3. Study Population

The source population included all outpatients aged 65 years or older who were seen at King Fahad University Hospital during the study period. Inclusion criteria included a documented diagnosis of type 2 diabetes and at least one of the following comorbidities recorded on or before the index date: ASCVD, heart failure, or CKD. In accordance with the 2025 ADA Standards of Care, patients meeting these comorbidity criteria were classified as guideline-eligible for SGLT2 inhibitor and/or GLP-1 receptor agonist therapy [8].

Diagnoses were obtained from EMR ICD-10 fields. The index encounter was defined as the most recent outpatient visit within the study period that met all inclusion criteria. Exclusions included type 1 diabetes; records missing key identifiers (MRN, date of birth, or encounter/dispense date); invalid dates (outside 1 June 2019–31 May 2023 or yielding age < 65 at index); and non-outpatient encounters. For specialty-specific estimates, we developed a patient-by-specialty analytic file. Each patient contributed no more than one observation per specialty (e.g., primary care, cardiology, internal medicine, other), representing the most recent visit in that specialty during the study period, following the method used by Dixon et al. [15].

### 4.4. Exposure and Outcomes

The exposure involved prescribing either an SGLT2 inhibitor (empagliflozin, dapagliflozin, canagliflozin, or ertugliflozin) or a GLP-1 receptor agonist (liraglutide, semaglutide, dulaglutide, exenatide, or lixisenatide) at any point during the study period, with pharmacy dispensing records serving as evidence that a prescription was issued. The analysis did not assess medication discontinuation. Patients who had initiated therapy prior to the study window but were no longer receiving treatment at the time of data capture were therefore not included. The primary outcome was the proportion of eligible patients who received either therapy during the study period. Secondary outcomes included prescribing rates by specialty, prescribing trends by calendar quarter and year, and patient-level factors associated with being prescribed these medications.

### 4.5. Specialty Attribution

Specialty-specific analyses used the patient-by-specialty dataset described above: each patient contributed one record per specialty (primary care, cardiology, internal medicine, other), representing the most recent visit in that specialty; a patient was considered “prescribed” for a specialty if any SGLT2 inhibitor/GLP-1 receptor agonist dispensing during the window was linked to care in that specialty, consistent with prior work [15].

### 4.6. Covariates

We included age, sex, treating specialty at the most recent visit (primary care, cardiology, internal medicine, other), comorbidity indicators for atherosclerotic cardiovascular disease, heart failure, and chronic kidney disease (ICD-10-based), and calendar time (year and quarter).

### 4.7. Statistical Analysis

Analyses were conducted at the patient level. Continuous variables were summarized as mean ± SD and categorical variables as counts (n) and percentages (%). The primary prescribing rate was defined as the proportion of eligible patients who received at least one SGLT2 inhibitor or GLP-1 receptor agonist during the study window. Specialty-specific rates were calculated from the patient-by-specialty dataset; the numerator was the number of eligible patients with at least one dispensing of either class during the window, and the denominator was the number of eligible patients with at least one outpatient visit in that specialty during the window. Each patient contributed one record per specialty based on the most recent visit. Yearly and quarterly trends were summarized descriptively as the proportion of eligible patients whose first dispensing occurred in each period; these summaries were not included in regression models.

Associations with prescribing were examined using logistic regression with prescription status as the dependent variable. Univariable models included age, sex, marital status, specialty, and comorbidity indicators for ASCVD, heart failure, and CKD. The multivariable model included age, sex, marital status, specialty, and the three comorbidity indicators. Regression results are presented as odds ratios with 95% confidence intervals, using two-sided *p* < 0.05 to define statistical significance. All analyses were performed in SAS 9.4 (SAS Institute, Cary, NC, USA).

## 5. Conclusions

In this real-world cohort of older adults with type 2 diabetes, overall use of SGLT2 inhibitors and GLP-1 receptor agonists was low at the population level. However, among individuals with documented ASCVD, heart failure, or chronic kidney disease, prescribing rates were high and largely consistent with guideline recommendations. These findings suggest that the primary barrier is not clinician reluctance to prescribe but rather incomplete recognition or documentation of high-risk comorbidities. Improving systematic cardiometabolic risk assessment and enhancing interdisciplinary care pathways may expand appropriate access to these therapies and support better cardiovascular and kidney outcomes in older adults.

## Figures and Tables

**Table 1:** Demographic and Clinical Characteristics of Older Adults (≥65 years) With Type 2 Diabetes in the Full Outpatient Cohort (N = 14,146) and the Guideline-Eligible Subgroup (N = 223).

Characteristic	Total Eligible (N = 223)	Prescribed SGLT2i/GLP-1 RA (N = 1052)	Not Prescribed (N = 13,094)
Age, mean ± SD	74.92 ± 6.23	73.55 ± 6.04	75.38 ± 7.74
Female, n (%)	94 (42.15%)	413 (39.40%)	5948 (49.28%)
Comorbidities:			
ASCVD, n (%)	209 (93.72%)	172 (1.22%)	706 (4.99%)
Heart failure, n (%)	9 (4.04%)	7 (0.05%)	26 (0.18%)
CKD, n (%)	8 (3.59%)	7 (0.05%)	40 (0.28%)

Abbreviations: ASCVD = atherosclerotic cardiovascular disease; CKD = chronic kidney disease; GLP-1 RA = glucagon-like peptide-1 receptor agonist; SD = standard deviation; SGLT2i = sodium–glucose cotransporter-2 inhibitor.

**Table 2:** (A) Overall Prescribing of GLP-1 Receptor Agonists or SGLT2 Inhibitors Among Older Adults (≥65 years) in the Full Outpatient Cohort (N = 14,146). (B) Prescribing of GLP-1 Receptor Agonists or SGLT2 Inhibitors Among Guideline-Eligible Older Adults (≥65 years) (N = 223).

(A)			
Outcome	Number Prescribed (N)	Total Cohort (N)	Prescribing Rate (%)
Any SGLT2 inhibitor or GLP-1 receptor agonist	1052	14,146	7.4%
**(B)**			
**Outcome**	**Number** **Prescribed (N)**	**Number** **Eligible (N)**	**Prescribing Rate Among Eligible (%)**
Any SGLT2 inhibitor or GLP-1 receptor agonist	186	223	83.4%

Abbreviations: GLP-1 = glucagon-like peptide-1; SGLT2 = sodium–glucose cotransporter-2. Eligibility based on guideline-recommended indications (see Section 4).

**Table 3:** Prescribing of GLP-1 Receptor Agonists or SGLT2 Inhibitors Among Guideline-Eligible Older Adults, Stratified by Comorbidity.

Comorbidity Subgroup	Number Eligible (N)	Number Prescribed (N)	Prescribing Rate (%)
ASCVD	209	172	85.29%
Heart Failure	9	7	77.77%
CKD	8	7	87.5%

Abbreviations: ASCVD = atherosclerotic cardiovascular disease; CKD = chronic kidney disease.

**Table 4:** Yearly and Quarterly Prescribing Rates of GLP-1 Receptor Agonists and SGLT2 Inhibitors Among Older Adults (2019–2023).

Year	Q1 N (%)	Q2 N (%)	Q3 N (%)	Q4 N (%)	Annual Total %
2019	243 (1.72%)	78 (0.55%)	58 (0.41%)	53 (0.37%)	432 (3.05%)
2020	56 (0.40%)	18 (0.13%)	15 (0.11%)	53 (0.37)	142 (1.00%)
2021	46 (0.33%)	39 (0.28%)	39 (0.28%)	69 (0.49)	193 (1.36%)
2022	47 (0.33%)	51 (0.36%)	51 (0.36%)	73 (0.52%)	222 (1.57%)
2023	46 (0.33%)	17 (0.12%)	-	-	63 (0.45%)

Abbreviations: Q1–Q4 = calendar year quarters.

**Table 5:** Prescribing of GLP-1 Receptor Agonists and SGLT2 Inhibitors by Specialty.

Specialty	Number Eligible (N)	Number Prescribed (N)	Prescribing Rate (%)
Medicine	16	11	68.75
Cardiology	70	31	44.28
Primary Care	122	52	42.62
Other Specialties *	15	4	26.66
Total	223	98	

* Other specialties include critical care, ear, nose, and throat (ENT), neurology, ophthalmology, psychiatry, and urology.

**Table 6:** Factors Associated with Prescribing of GLP-1 Receptor Agonists and SGLT2 Inhibitors.

Variables	Groups	Univariate LR			Multivariate LR		
		ODDS	95% CI	*p*	ODDS	95% CI	*p*
Age		0.964	0.955–0.974	<0.001	0.957	0.947–0.968	<0.001
Specialty	Primary Care (Ref.)	-	-	<0.001	-	-	<0.001
	Cardiology	1.723	1.195–2.484	0.004	1.572	1.059–2.331	0.025
	Medicine	1.419	1.112–1.811	0.005	1.448	1.116–1.878	<0.005
	Other Specialties	0.399	0.314–0.507	<0.001	0.399	0.309–0.516	<0.001
Marital Status	Married (Ref.)	-	-	0.000	-	-	-
	Unmarried	0.562	0.473–0.667	<0.001	0.663	0.551–0.798	<0.001
Sex	Male (Ref.)	-	-	0.000	-	-	<0.001
	Female	0.695	0.608–0.795	<0.001	0.715	0.622–0.820	<0.001

Abbreviations: CI = confidence interval; LR = logistic regression; Ref. = reference category.

**Table 7:** Prescribing Rates of GLP-1 Receptor Agonists and SGLT2 Inhibitors by Specialty in Patients with ASCVD.

Variables	Groups	Univariate LR			Multivariate LR		
		ODDS	95% CI	*p*	ODDS	95% CI	*p*
Age		0.988	0.956–1.021	0.477	0.999	0.960–1.040	0.975
Specialty	Primary Care (Ref.)	-	-	0.002	-	-	0.328
	Cardiology	1.741	0.415–7.310	0.449	1.445	0.277–7.534	0.662
	Medicine	1.039	0.374–2.892	0.941	0.979	0.312–3.074	0.970
	Other Specialties	0.409	0.151–1.111	0.080	0.098	0.127–1.192	0.098
Marital Status	Married (Ref.)	-	-	-	-	-	-
	Unmarried	0.939	0.500–1.763	0.844	1.139	0.579–2.242	0.706
Sex	Male (Ref.)	-	-	-	-	-	-
	Female	0.581	0.336–1.1.004	0.050	0.555	0.371–0.972	0.040

Abbreviations: CI = confidence interval; LR = logistic regression; Ref. = reference category.

**Table 8:** Prescribing Rates of GLP-1 Receptor Agonists and SGLT2 Inhibitors by Specialty in Patients with Heart Failure.

Variables	Groups	Univariate LR			Multivariate LR		
		ODDS	95% CI	*p*	ODDS	95% CI	*p*
Age		0.894	0.968–1.114	0.373	0.931	0.711–1.218	0.602
Specialty	Primary Care (Ref.)	-	-	0.356	-	-	0.827
	Cardiology	0.000	0.000–0.000	0.810	0.890	0.146–13.730	0.763
	Medicine	0.000	0.000–0.000	1.000	515,924,539.332	0.000–0.000	1.000
	Other Specialties	0.261	0.000–0.000	0.999	562,657,743.049	0.000–0.000	0.999
Marital Status	Married (Ref.)	-	-	-	-	-	-
	Unmarried	0.999	0.000–0.000	0.999	0.999	0.000–0.000	0.999
Sex	Male (Ref.)	-	-	-	-	-	-
	Female	0.999	0.000–0.000	0.999	0.999	0.000–0.000	0.999

Abbreviations: CI = confidence interval; LR = logistic regression; Ref. = reference category.

**Table 9:** Prescribing Rates of GLP-1 Receptor Agonists and SGLT2 Inhibitors by Specialty in Patients with Chronic Kidney Disease.

Variables	Groups	Univariate LR			Multivariate LR		
		ODDS	95% CI	*p*	ODDS	95% CI	*p*
Age		0.683	0.291–1.604	0.381	0.442	0.078–2.508	0.357
Specialty	Primary Care (Ref.)	-	-	-	-	-	-
	Cardiology	1.000	0.000–0.000	1.000	5.273	0.161–8.649	1.000
	Medicine	-	-	-	-	-	-
	Other Specialties	-	-	-	-	-	-
Marital Status	Married (Ref.)	-	-	-	-	-	-
	Unmarried	0.000	0.000–0.000	0.999	0.000	0.000–0.000	0.999
Sex	Male (Ref.)	-	-	-	-	-	-
	Female	0.000	0.000–0.000	0.999	5,310,170.02	0.000–0.000	0.999

Abbreviations: CI = confidence interval; LR = logistic regression; Ref. = reference category.

## Data Availability

The original contributions presented in this study are included in the article. Further inquiries can be directed to the corresponding author.

## Supplementary Materials

The following supporting information can be downloaded at https://www.mdpi.com/article/10.3390/ph19010009/s1: Supplementary Table S1: Distribution of prescribed SGLT2 inhibitors and GLP-1 receptor agonists stratified by cardiometabolic comorbidities (ASCVD, heart failure, and chronic kidney disease) among older adults.

## Abbreviations

The following abbreviations are used in this manuscript:

ASCVD	Atherosclerotic Cardiovascular Disease
CKD	Chronic Kidney Disease
GLP-1 RA	Glucagon-like Peptide-1 Receptor Agonist
SGLT2i	Sodium–Glucose Cotransporter-2 Inhibitor
