# Supplementary material for: Real-World Prescribing Patterns of SGLT2 Inhibitors and GLP-1 Receptor Agonists in Older Adults with Type 2 Diabetes and Cardiometabolic Disease
Source: Pharmaceuticals (Basel). 2025 Dec 20;19(1):9. doi: 10.3390/ph19010009 (PMC12845426; doi:10.3390/ph19010009)
Supplement: Supplementary file 1 [file pharmaceuticals-19-00009-s001.zip › pharmaceuticals-4059886-supplementary.pdf]

Supplementary Table S1. Distribution of prescribed SGLT2i and GLP-1 RA by comorbidity.

Medication	Drug Class	Total Prescribed (N)	ASCVD (N)	HF (N)	CKD (N)
**Dapagliflozin**	SGLT2 inhibitor	768	135	6	7
**Dulaglutide**	GLP-1 receptor agonist	237	36	1	0
**Liraglutide 6 mg/mL injection**	GLP-1 receptor agonist	47	7	0	0
**Total**	–	**1,052**	178	7	7
